# Vitexicarpin Induces Apoptosis and Inhibits Metastatic Properties via the AKT-PRAS40 Pathway in Human Osteosarcoma

**DOI:** 10.3390/ijms25073582

**Published:** 2024-03-22

**Authors:** Hyung-Mun Yun, Hyun Sook Kwon, Joon Yeop Lee, Kyung-Ran Park

**Affiliations:** 1 Department of Oral and Maxillofacial Pathology, School of Dentistry, Kyung Hee University, Seoul 02447, Republic of Korea; 2 National Development Institute for Korean Medicine, Gyeongsan 38540, Republic of Korea; iamsook@nikom.or.kr (H.S.K.); chool9090@nikom.or.kr (J.Y.L.); 3 Gwangju Center, Korea Basic Science Institute (KBSI), Gwangju 61751, Republic of Korea

**Keywords:** AKT, apoptosis, autophagy, invasion, osteosarcoma, PRAS40, Vitexicarpin

## Abstract

Osteosarcoma, which has poor prognosis after metastasis, is the most common type of bone cancer in children and adolescents. Therefore, plant-derived bioactive compounds are being actively developed for cancer therapy. *Artemisia apiacea* Hance ex Walp. is a traditional medicinal plant native to Eastern Asia, including China, Japan, and Korea. Vitexicarpin (Vitex), derived from *A. apiacea,* has demonstrated analgesic, anti-inflammatory, antitumour, and immunoregulatory properties; however, there are no published studies on Vitex isolated from the aerial parts of *A. apiacea.* Thus, this study aimed to evaluate the antitumour activity of Vitex against human osteosarcoma cells. In the present study, Vitex (>99% purity) isolated from *A. apiacea* induced significant cell death in human osteosarcoma MG63 cells in a dose- and time-dependent manner; cell death was mediated by apoptosis, as evidenced by the appearance of cleaved-PARP, cleaved-caspase 3, anti-apoptotic proteins (Survivin and Bcl-2), pro-apoptotic proteins (Bax), and cell cycle-related proteins (Cyclin D1, Cdk4, and Cdk6). Additionally, a human phosphokinase array proteome profiler revealed that Vitex suppressed AKT-dependent downstream kinases. Further, Vitex reduced the phosphorylation of PRAS40, which is associated with autophagy and metastasis, induced autophagosome formation, and suppressed programmed cell death and necroptosis. Furthermore, Vitex induced antimetastatic activity by suppressing the migration and invasion of MMP13, which is the primary protease that degrades type I collagen for tumour-induced osteolysis in bone tissues and preferential metastasis sites. Taken together, our results suggest that Vitex is an attractive target for treating human osteosarcoma.

## 1. Introduction

Osteosarcoma, caused by osteoblasts of mesenchymal origin that deposit an immature osteoid matrix, is the most common malignant bone cancer in children and adolescents [1,2]. Symptoms of osteosarcoma, such as pain, swelling, decreased joint motion, and fracture, may appear at tumour sites [3]. The survival rate for cases where osteosarcoma is localised and has not spread to other parts of the body is 70%, but when it metastasises to the lungs or other bones at the time of diagnosis, the long-term survival rate decreases significantly to approximately 30% [4]. Neoadjuvant chemotherapy, surgical resection, and adjuvant chemotherapy are currently recommended for osteosarcoma treatment [1]. However, continued best studies have not resulted in a discernible improvement in the five-year survival rate of patients [5]. Further, the number of patients diagnosed with osteosarcoma in the last few decades indicates that current therapeutic approaches are inadequate. Therefore, the identification of novel therapeutic compounds with biological effects against osteosarcoma is critically needed.

Substantial evidence suggests that the disease is associated with the deregulation of various intracellular signalling pathways, most notably the AKT signalling pathway, which plays a critical role in the biological activities and homeostasis of cells [6,7]; AKT signalling is essential for a wide range of physiological and pathological processes [8]. Specifically, the route plays a major role in practically all human cancer types as an oncogenic pathway; the deregulation of AKT signalling plays an important role in proliferation, apoptosis, invasion, and carcinogenesis [9,10,11,12]. Thus, AKT signalling has been targeted by several small-molecule drugs to improve the survival of patients with osteosarcoma.

Recently, the remarkable biological activities of species of the globally prevalent genus *Artemisia* (family Asteraceae) have garnered significant attention [13]. Of these, *Artemisia apiacea* Hance ex Walp. is one of the traditional medicinal plants most widely used for the treatment of malaria, jaundice, and eczema in Eastern Asia, including in China, Japan, and Korea [14]. Numerous studies have investigated the anti-inflammatory activities of *A. apiacea* extract, and the immunosuppressive, anti-allergic, anti-pyretic, anti-bacterial, and anti-parasitic activities of *A. apiacea* [14,15,16]. A flavonoid called Vitexicarpin (Vitex), which is extracted from *A. apiacea*, has shown cytotoxic, anti-inflammatory, anti-tumour, anti-angiogenic, analgesic, and immunomodulatory effects [17]. However, the biological and pharmacological effects of Vitex on osteosarcoma have not been reported.

Here, for the first time, we isolated Vitex from the aerial parts of *A. apiacea* plants to explore the potential activities and biological mechanisms of Vitex in osteosarcoma. The aim of this study was to investigate the anti-osteosarcoma effects of Vitex with >99% purity in vitro in human osteosarcoma cells.

## 2. Results

### 2.1. Purification and Characterization of Vitex from A. apiacea

**Figure 1:**
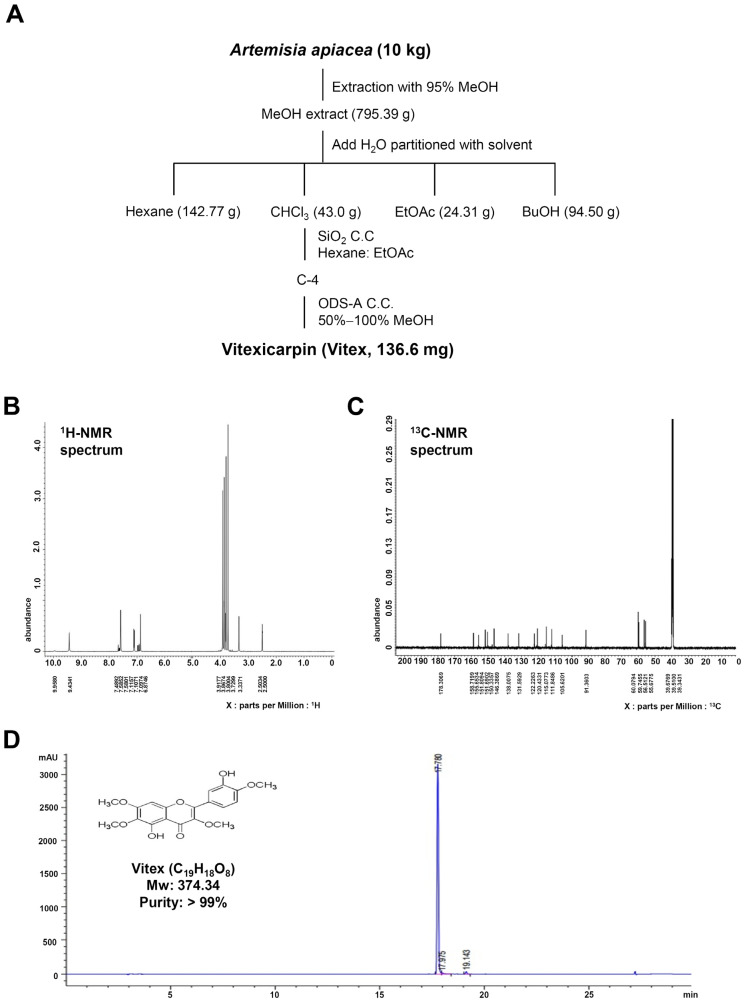
Purification of Vitexicarpin (Vitex) from aerial parts of *Artemisia apiacea*. (**A**) Procedure for the isolation of Vitex. (**B**,**C**) ^1^H-NMR (500 MHz, DMSO-*d*_6_) (**B**) and ^13^C-NMR (100 MHz, CD_3_OD) (**C**) spectra of Vitex. (**D**) HPLC analysis of purified Vitex. The inset shows Vitex’s chemical structure, molecular formula, and purity.

The procedure illustrated in Figure 1A was used to separate Vitex from 10 kg of *Artemisia apiacea* (Figure 1A).

^1^H-nuclear magnetic resonance (NMR) (500 MHz, DMSO-*d*_6_) assign data: δ 9.95 (1H, s, 5-OH); 9.43 (1H, s, 3′-OH); 7.59 (2H, dd, *J* = 2.0, 8.0 Hz, H-2′, 6′); 7.11 (1H, d, *J* = 8.0 Hz, H-5′); 6.87 (1H, s, H-5); 3.91 (3H, s, 7-OCH_3_); 3.86 (3H, s, 4′-OCH_3_); 3.80 (3H, s, 3-OCH_3_); 3.72 (3H, s, 6-OCH_3_) (Figure 1B). 

^13^C-NMR (125 MHz, DMSO-*d*_6_) assign data: δ 55.6 (4′-OCH_3_); 56.5 (7-OCH_3_); 59.7 (3-OCH_3_); 60.0 (6-OCH_3_); 91.3 (C-8); 105.6 (C-10); 111.8 (C-5′); 115.0 (C-2′); 120.4 (C-6′); 122.2 (C-1′); 131.5 (C-6); 138.0 (C-3); 146.3 (C-4′); 150.3 (C-3′); 151.6 (C-5); 151.8 (C-9); 155.6 (C-2); 158.7 (C-7); 178.3 (C-4) (Figure 1C). The resulting Vitex was a pale-yellow powder with a molecular formula of C_19_H_18_O_8_ and a purity of >99%. The high-performance liquid chromatography (HPLC) results and chemical structure of Vitex are shown in Figure 1D.

### 2.2. Vitex Reduces Cell Viability in Human Osteosarcoma Cells

**Figure 2:**
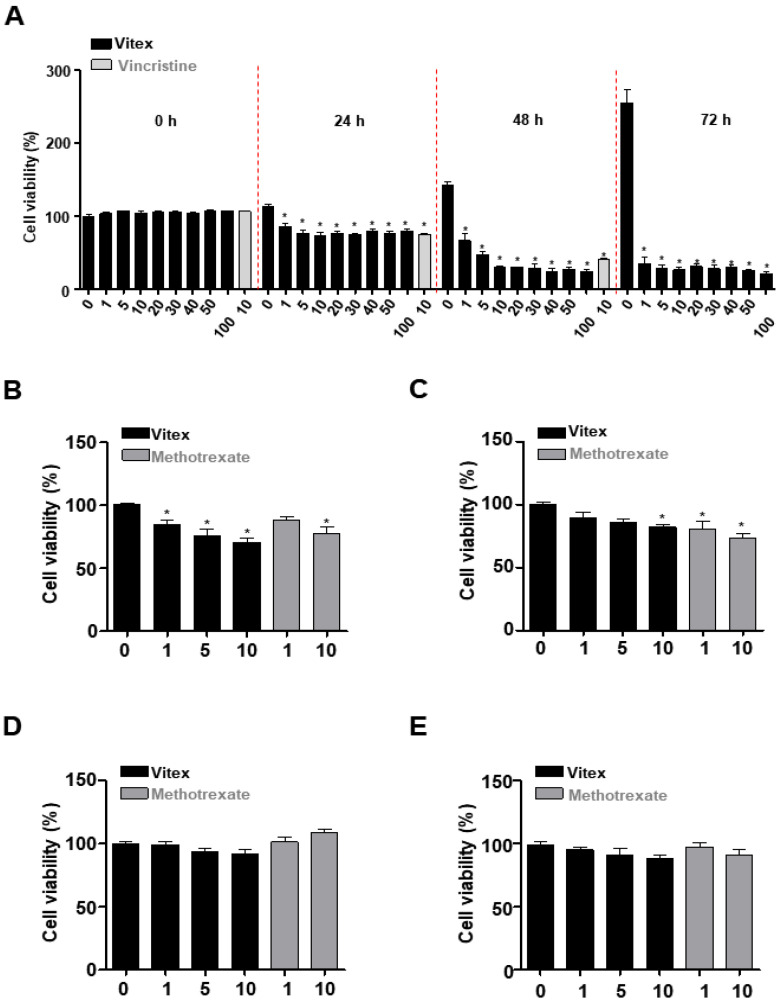
Effects of Vitex on cell viability in human osteosarcoma cells. (**A**) Cell viability (%) was detected using an MTT assay after Vitex treatment at concentrations ranging from 0 to 100 μM, or 10 μM of Vincristine treatment for 0 to 72 h in human MG63 osteosarcoma cells. (**B**,**C**) After Vitex or Methotrexate treatment for 24 h, the cell viability (%) was detected in human MG63 (**B**) and SJSA1 (**C**) osteosarcoma cells using an MTT assay. (**D**,**E**) After Vitex or Methotrexate treatment for 24 h, the cell viability (%) was detected via an MTT assay in MSCs (**D**) and pre-osteoblasts (**E**). Asterisks indicate statistical significance (* *p* < 0.05); data represent the results of three experiments.

To investigate the anti-osteosarcoma activities of Vitex extracted from *A. apiacea* Hance ex Walp., we screened its activities at various concentrations ranging from 1 to 100 μM for 0 to 72 h. Vincristine was used as a positive control because it is a well-known chemotherapeutic drug used to treat various malignant tumours [18,19]. The cell viability was analysed using an MTT assay, and the results indicated that Vitex significantly reduced the viability (%) of human osteosarcoma cells in a dose- and time-dependent manner (Figure 2A). In subsequent experiments, the human osteosarcoma cells were treated with 1, 5, and 10 μM of Vitex. When comparing the viability of Vitex and Vincristine at 10 μM, the inhibitory effect of Vitex against human osteosarcoma cells exceeded that of vincristine. 

To further examine the effect of Vitex on the viability of p53 mutant-type osteosarcoma MG63 cells and p53 wild-type osteosarcoma SJSA1 cells treated with an anti-osteosarcoma drug (Methotrexate), Vitex was administered for 24 h, and the cell viability was analysed using an MTT assay. The results showed that Vitex significantly reduced the viability of MG63 cells to a greater extent than that of SJSA1 cells (Figure 2B,C). Next, toxicity tests were performed on C3H/10T1/2 mesenchymal stem cells (MSCs) and MC3T3-E1 pre-osteoblasts. Vitex was not as highly toxic to normal cells as the known anti-osteosarcoma drug Methotrexate (Figure 2D,E). These results suggest that Vitex exerts an inhibitory effect on human osteosarcoma cells.

### 2.3. Vitex Induces Programmed Apoptotic Cell Death in Human Osteosarcoma Cells

**Figure 3:**
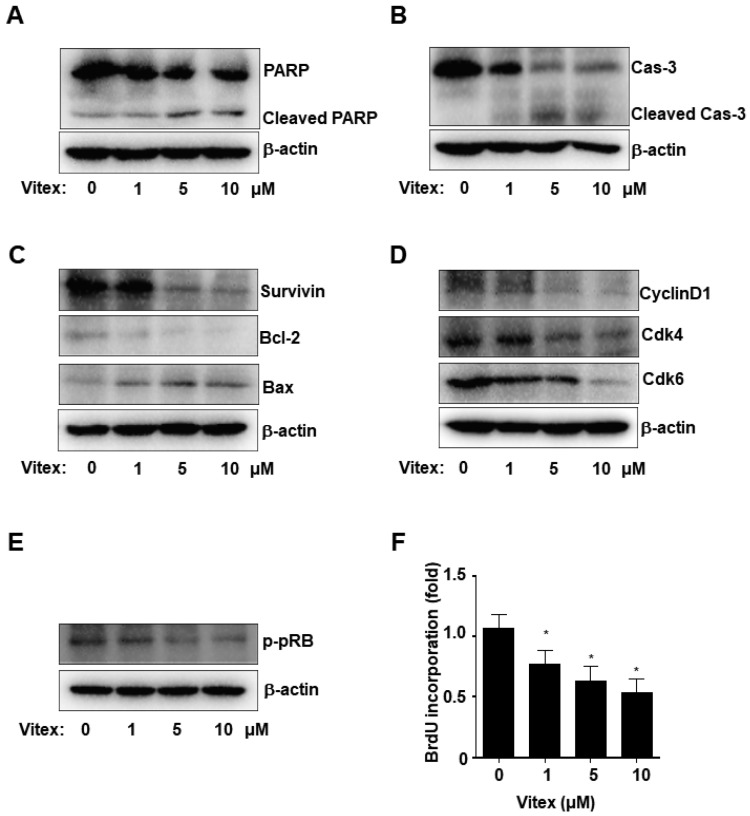
Effects of Vitex on apoptosis in the human osteosarcoma cells. (**A**–**C**) Western blotting of PARP and cleaved PARP (**A**); caspase 3 and cleaved caspase 3 (**B**); and Survivin, Bcl-2, and Bax protein levels (**C**). (**D**,**E**) Western blotting of cyclinD1, Cdk4, and Cdk6 (**D**) protein levels, as well as pRB phosphorylation levels (**E**). The amount of β-actin was detected as a loading control in the same sample. (**F**) BrdU incorporation assay results. Asterisks indicate statistical significance (* *p* < 0.05); data represent the results of three experiments.

To investigate whether programmed apoptotic cell death is a mechanism of the inhibitory effects of Vitex, we analysed apoptosis via the caspase cascade using western blotting. Vitex treatment enhanced the amount of cleaved PARP and caspase-3 products, and decreased the amount of full-length PARP and caspase-3 proteins in a dose-dependent manner (Figure 3A,B). Second, we assessed the amounts of pro- and anti-apoptotic proteins and found that Vitex treatment decreased the levels of Survivin and Bcl-2 proteins while increasing that of Bax proteins (Figure 3C). Third, we detected the cell cycle-related proteins required for cell proliferation and death. Fourth, Vitex treatment downregulated Cyclin D1, as well as the cyclin-dependent kinase 4 (Cdk4) and Cdk6 proteins (Figure 3D). We also detected the phosphorylation of pRB, a downstream target of CyclinD1 and CDK4/6, and found that Vitex treatment decreased pRB phosphorylation (Figure 3E). Additionally, bromodeoxyuridine (BrdU) is frequently incorporated during DNA synthesis for cell cycle analysis; therefore, we also validated the effect of Vitex on cell proliferation and the cell cycle using a BrdU incorporation assay, and found that Vitex treatment suppressed BrdU incorporation (Figure 3F). These results suggest that the inhibitory effects of Vitex are mediated by apoptosis in human osteosarcoma cells.

### 2.4. Vitex Inhibits the AKT-PRAS40 Pathway in Human Osteosarcoma Cells

**Figure 4:**
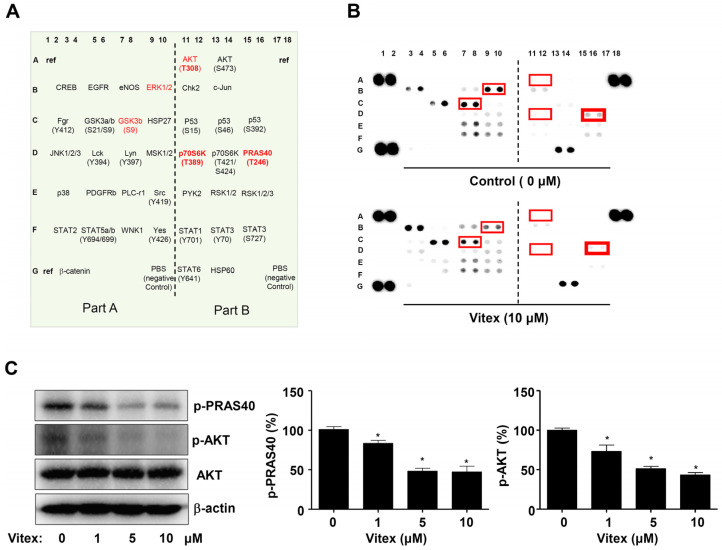
Effects of Vitex on intracellular signalling proteins in human osteosarcoma cells. (**A**) The proteome profiler human phospho-kinase array contains 37 different kinases, including three references and two PBS negative control kinases. Red text: Proteins with large differences. (**B**) Vitex treatment shows the relative expression of the phosphorylation profiles of 37 different kinases. Double spots with large differences are marked with red rectangles. (**C**) Western blots of phospho-PRAS40 (p-PRAS40), phospho-AKT (p-AKT), AKT, and β-actin levels. The percentage change is depicted in a bar graph. The amount of β-actin was detected as a loading control in the same sample. Asterisks indicate statistical significance (* *p* < 0.05); data represent the results of three experiments.

To explore how Vitex induces apoptotic cell death through intracellular signalling proteins, we screened the response to Vitex using a proteome profiler human phospho-kinase array consisting of 37 different kinases. We identified a significant decrease in the phosphorylation of AKT signalling-related proteins, including AKT(T308), a 40 kDa proline-rich Akt substrate (PRAS40), p-70S6K(T389), GSK3β(S9), and ERK1/2 (Figure 4A,B). The phosphorylation of PRAS40 by AKT blocks mTORC1 inhibitory function, and PRAS40 competes with the mTOR downstream substrates 4E-BP1 and p70S6K by binding to Raptor. Then, p70S6K activates functions related to cell proliferation and the cell cycle. Consistent with the screening results, western blotting revealed a significant decrease in the phosphorylation of AKT and PRAS40 (Figure 4C,D). Additionally, we confirmed a decrease in STAT3 phosphorylation, one of the targets of Vitex (Supplementary Figure S1). These results suggest that the AKT-PRAS40 pathway is involved in the inhibitory effects of Vitex on human osteosarcoma cells.

### 2.5. Vitex Inhibits the AKT-PRAS40 Pathway-Associated Autophagy and Suppresses Necroptosis in Human Osteosarcoma Cells

**Figure 5:**
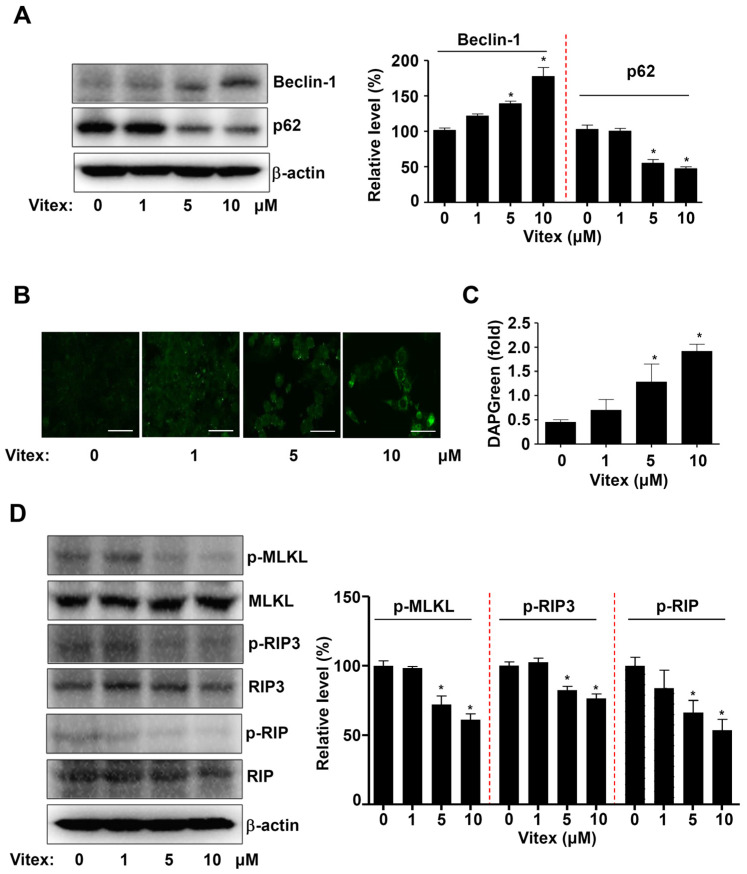
Effects of Vitex on autophagy and necroptosis in human osteosarcoma cells. (**A**) Western blotting of Beclin1, p62, and β-actin levels. (**B**) Fluorescence microscopy to assess DAPGreen-stained autophagosomes in human osteosarcoma cells. Scale bar: 50 μm. (**C**) The DAPGreen (fold) is depicted in a bar graph. (**D**) Western blotting of phospho-MLKL (p-MLKL), MLKL, phospho-RIP3 (p-RIP3), RIP3, phospho-RIP (p-RIP), RIP, and β-actin levels. The amount of β-actin was detected as a loading control in the same sample. Asterisks indicate statistical significance (* *p* < 0.05); data represent the results of three experiments.

Because the AKT-PRAS40 pathway is a representative autophagy-regulating protein, we next investigated whether Vitex could regulate autophagosome formation in human osteosarcoma cells. We analysed the effects of vitex on the autophagy-related proteins Beclin-1 and p62 in vitro using western blotting and found that Vitex treatment increased Beclin-1 protein levels but decreased those of p62 protein (Figure 5A). To further validate the effects of Vitex on autophagosome formation, autophagic vacuole formation was analysed using DAPGreen and an immunofluorescence assay. The observation showed that Vitex treatment increased autophagosome formation (Figure 5B,C). 

Necroptosis is another type of programmed cell death that differs from, and competes, with apoptosis. Therefore, we investigated the effect of Vitex on necroptosis in human osteosarcoma cells. The key components of the necroptosis machinery, namely receptor-interacting serine/threonine-protein kinase (RIP), RIP3, and mixed-lineage kinase domain-like pseudokinase (MLKL), were detected using western blotting. We found that Vitex treatment decreased the phosphorylation of RIP, RIP3, and MLKL (Figure 5D). Overall, our findings suggest that Vitex induces AKT-PRAS40 pathway-associated autophagy, but blocks necroptosis in human osteosarcoma cells.

### 2.6. Vitex Exerts Anti-Metastatic Effects through the Inhibition of Migration and Invasion in Human Osteosarcoma Cells

**Figure 6:**
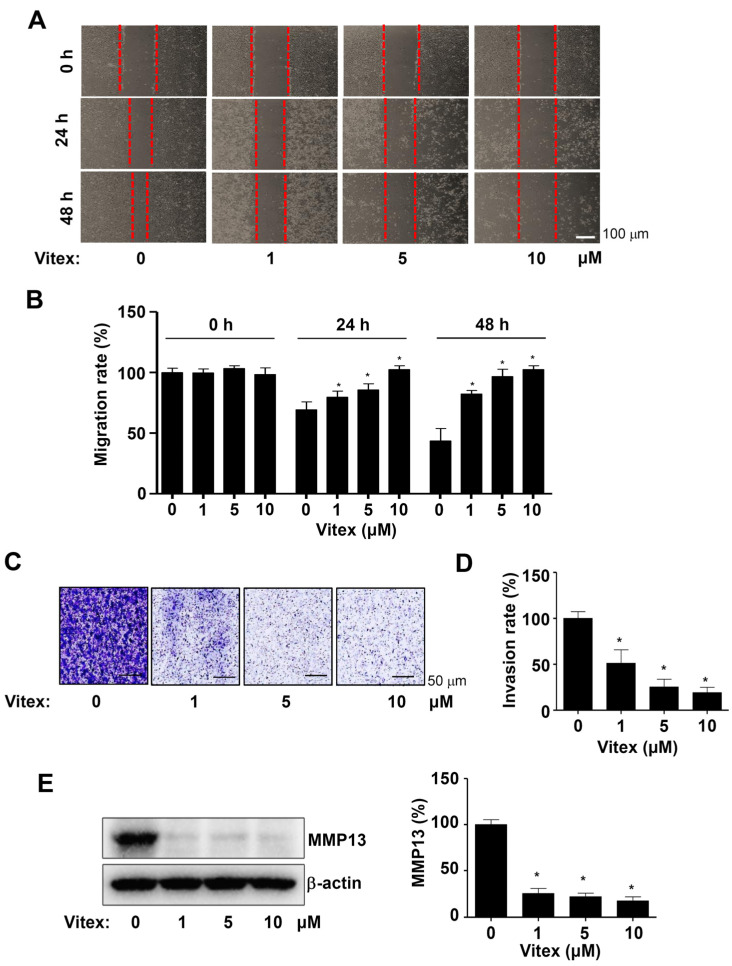
Effects of Vitex on cell migration and invasion of human osteosarcoma cells. (**A**,**B**) Migrative cells were detected using a wound-healing assay, and images were captured using a light microscope. Red dotted lines: migration region. Scale bar: 100 μm (**A**). The migration rate (%) is depicted in a bar graph (**B**). (**C**,**D**) Invasive cells were detected using Boyden chamber assays, and the images were obtained using a light microscope. Scale bar: 50 μm (**C**). The invasion rate (%) is depicted in a bar graph (**D**). (**E**) Western blots of MMP13 and β-actin levels. The amount of β-actin was detected as a loading control in the same sample. Asterisks indicate statistical significance (* *p* < 0.05); data represent the results of three experiments.

Finally, to examine whether Vitex has anti-metastatic effects, we investigated the migration of human osteosarcoma cells using a wound-healing migration assay. Vitex treatment significantly reduced migration into the wound areas at 24 and 48 h compared to that in untreated control cells (Figure 6A,B). Next, we performed a Boyden chamber assay to observe cell invasion and extracellular matrix degradation during metastasis. As shown in Figure 6C,D, Vitex treatment significantly attenuated penetration and transmigration across the Matrigel-coated membrane compared to those in the untreated control cells (Figure 6C,D). Additionally, Vitex treatment for 24 h decreased the protein levels of matrix metalloproteinase 13 (MMP13), which degrades the extracellular matrix during metastasis (Figure 6E). Our results suggest that Vitex inhibits the metastatic properties of human osteosarcoma cells.

## 3. Discussion

A complex cascade of signalling pathways is involved in multifactorial and multistage diseases such as cancer. Despite the great heterogeneity of the various human tumour types, many cancers share eight characteristics known as the “hallmarks of cancer” [20,21,22], including uncontrolled proliferation, resistance to apoptosis, the evasion of growth suppression, induction and access to the vasculature, the activation of invasion and metastasis, the avoidance of immune destruction, and continuous proliferative signalling [21,22]. According to this theory, the malignant transformation of normal cells and benign tumours evades apoptosis through an imbalance between programmed cell death and proliferation. Natural compounds have drawn much interest in the development of medications to treat various cancers, with a focus on cell death [23,24,25]. Natural compounds, which have been used in traditional medicine for centuries, are often safer and more affordable than chemically manufactured pharmaceuticals [23,24,25]. In the present study, we demonstrated the effects of Vitex, isolated from *A. apiacea*, on apoptotic cell death and its antimetastatic properties through the AKT-PRAS40 pathway in human osteosarcoma cells.

Apoptotic cell death is primarily mediated by caspases, a family of cysteine proteases. The Bcl-2 family, which includes Bax and Bcl-2 proteins, and inhibitors of the apoptosis protein family, such as survivin, control caspase cascades as apoptotic regulatory proteins [26,27]. Pro-apoptotic Bax proteins, which form macropores in the outer mitochondrial membrane, facilitate the escape of cytochrome C from the mitochondria, while anti-apoptotic Bcl-2 proteins prevent apoptosis by inhibiting cytochrome C translocation and blocking caspase activation and the apoptotic process [28,29]. Survivin, as a caspase inhibitor, inhibits apoptosis by suppressing the caspase cascade and cleavage mediated by caspases [30]. We demonstrated that Vitex increased the active form of caspase-3, decreased levels of Bcl-2 and survivin proteins, and increased Bax protein levels in human osteosarcoma cells. Moreover, active caspase-3 induces the cleavage of PARP products, which lose their DNA repair activity, leading to DNA strand-break signals and cell cycle arrest; thus, PARP cleavage is a biochemical feature of apoptosis [31,32,33]. In this study, we demonstrated that Vitex increased PARP cleavage and reduced the protein expressions of Cdk4, Cdk6, and Cyclin D1. Of these, Cyclin D1 induces the transition from the G1 to S phase through complex formation with Cdk4 and Cdk, leading to tumour growth. These proteins are overexpressed at high frequencies in various human cancers [34,35,36]. Therefore, our results demonstrated that Vitex exerts anti-osteosarcoma effects by inducing caspase-dependent apoptotic cell death and suppressing cell growth in human osteosarcoma cells.

Previous studies have reported that AKT signalling proteins inhibit apoptotic cell death and increase cell cycle progression and metastasis through various pathways in human osteosarcoma cells [10,37,38,39]. The hyperactivation and overexpression of AKT signalling proteins contribute to tumorigenesis, proliferation, apoptosis, invasion, apoptosis, and metastasis in human osteosarcoma cells [40]. Thus, targeting AKT signalling proteins is a promising treatment strategy for osteosarcoma. In the present study, we demonstrated that Vitex inhibits the phosphorylation of AKT and its signalling proteins. Increased PRAS40 phosphorylation has been observed in malignant tumours and is associated with poor patient survival [41,42]. PRAS40 is a negative regulator of the mammalian target of rapamycin complex 1 (mTORC1), which is phosphorylated by AKT [43]. AKT-mediated PRAS40 phosphorylation increases cell growth, inhibits apoptotic cell death, increases metastasis, and contributes to tumorigenesis; thus, PRAS40 phosphorylation may be a novel biomarker or therapeutic target for tumour treatment [42]. In summary, AKT signalling has been targeted by several small-molecule drugs to improve the survival of patients with osteosarcoma. Previous studies have shown that by inhibiting the PI3K/AKT/mTOR pathway, Vitex induces apoptosis and cell cycle arrest in non-small cell lung cancers and glioblastoma [44]. In the present study, we demonstrated for the first time that Vitex decreases the phosphorylation of PRAS40 via the AKT signalling pathway in human osteosarcoma cells. These findings suggest that the anti-osteosarcoma effects of Vitex are mediated by intracellular mechanisms involving the AKT-PRAS40 pathway. 

Autophagy, a fundamental cellular process that involves lysosomal degradation through the removal and recycling of cellular molecules, including nucleic acids, proteins, lipids, and organelles, regulates cell survival and death [45]. Previous studies have shown that the crosstalk between apoptosis and autophagy inhibits tumorigenesis and metastasis [46,47], and that the inhibition of AKT signalling leads to autophagy and cell death in human osteosarcoma cells [48,49]. Here, we found that Vitex suppressed AKT and PRAS40 phosphorylation and enhanced autophagy through autophagosome formation in human osteosarcoma cells; autophagy was inhibited by the increased PRAS40 phosphorylation of AKT [50]. AKT signalling is also involved in another type of programmed cell death called necroptosis [51,52], which is a newly described caspase-independent form of regulated necrosis [53,54]. Usually, apoptosis processes prevent necroptosis via the RIP1-RIP3-MLKL signalling pathway [55,56]. In the present study, we demonstrated that Vitex inhibited the RIP1-RIP3-MLKL signalling pathway in human osteosarcoma cells, leading to increased apoptosis and reduced necroptosis. Thus, our findings suggest that Vitex exerts its anti-osteosarcoma effects by inducing autophagy and apoptosis via the AKT-PRAS40 pathway.

Osteosarcoma cells can metastasise to the lungs and, in some cases, to the bones or lymph nodes, and metastatic osteosarcoma is associated with decreased long-term survival [4]. Patients with metastases exhibit elevated PRAS40 mRNA expression [57], as PRAS40 enhances cancer cell growth by suppressing apoptosis and metastasis [42]. Previous studies have shown that PRAS40 downregulation increases the migration and invasion of head and neck squamous cell carcinoma cells [58]. Metastatic osteosarcoma cells migrate away from the primary tumour and invade neighbouring tissues during the initial stages of metastasis [59]. Additionally, the metastasis of osteosarcoma cells is associated with AKT-mediated GSK3β phosphorylation and ERK1/2 phosphorylation [59]. In this study, we found that Vitex suppressed AKT and the expression of its downstream target proteins PRAS40, GSK3β and ERK1/2. In the present study, we demonstrated that Vitex suppressed cell migration in a wound-healing assay, inhibited the invasion of human osteosarcoma cells and suppressed the expression of MMP-13 in human osteosarcoma cells. The increased invasion and lung metastasis of human osteosarcoma cells are attributed to elevated MMP-13 expression and release [60]. Therefore, our findings suggest that Vitex exerts anti-osteosarcoma effects on the metastatic properties of human osteosarcoma.

In conclusion, osteosarcoma is a potentially fatal disease that often reduces the quality of life of patients. Despite the recent advances in diagnostics and therapeutics, limitations that can be addressed by developing new bioactive compounds still exist. The current study is the first to present evidence that Vitex isolated from *A. apiacea* reduces cell migration, invasion, and survival, while promoting apoptotic cell death and autophagy, specifically by inhibiting the intracellular AKT-PRAS40 pathway in an in vitro human osteosarcoma cell system. While in vivo studies should be performed in the future, our findings suggest that Vitex is a promising bioactive compound for the chemotherapeutic treatment of human osteosarcoma. 

## 4. Materials and Methods

### 4.1. Plant Material and General Proceduresl

The aerial parts of *Artemisia apiacea* Hance ex Walp. (Origin: Yeongcheon-si, Gyeongsangbuk-do, Republic of Korea) were purchased from the commercial herbal medicine market. The P310 voucher specimen has been deposited in the Natural Products Bank at the National Institute for Korean Medicine Development (NIKOM). The nuclear magnetic resonance (NMR) spectra were obtained on a Jeol ECX-500 spectrometer (JEOL Ltd., Tokyo, Japan) operating at ^1^H (500 MHz) and ^13^C (125 MHz). The electron ionization mass spectrometer (EI-MS) data were obtained using the micromass spectrum (AUTOSPEC, Glasgow, UK). High-performance liquid chromatography (HPLC) was performed using Agilent 1200 series (Agilent Technologies, Santa Clara, CA, USA). Column chromatography (CC) was conducted using silica gel (70–230 mesh; Merck, Darmstadt, Germany).

### 4.2. Isolation of the Active Compound from A. apiacea

The aerial parts of *A. apiacea* (10 kg) were extracted with 100% MeOH (3 × 30 L) at room temperature. The combined extracts were concentrated under reduced pressure, and the residue (795.3 g) was partitioned into H_2_O and extracted with n-Hexane, CHCl_3_, and BuOH. The CHCl_3_ fraction (43.0 g) was subjected to open-column chromatography over silica gel (n-Hexane: EtOAc from 30:1 to 0:1) to give eleven fractions (A−K). Fraction H (9.1 g) was purified using a RP-C18 column with 60% MeOH to yield six subfractions (H-1~H-6). Fractions H-5 (0.8 g) were subjected to repeated Sephadex LH-20 column chromatography with 100% MeOH to isolate 136.6 mg of Vitexicarpin (Vitex, pale-yellow powder).

### 4.3. Cell Culture

Human osteosarcoma MG63 cells (#CRL-1427), isolated from the bone of a 14-year-old male patient with osteosarcoma, were obtained from the ATCC (Manassas, VA, USA). The cells were cultured in Dulbecco’s modified Eagle medium (WELGEME, Inc., Seoul, Republic of Korea) at 37 °C, 95% air, and 5% CO_2_. The p53 wild-type osteosarcoma cells (SJSA1, (#CRL-2098), MSCs (C3H/10T1/2, #CCL-226), and pre-osteoblasts (MC3T3-E1, #CRL-2593) were obtained from the ATCC (Manassas, VA, USA).

### 4.4. Cell Viability Analysis

Cell viability was analysed using the 3-[4,5-dimethylthiazol-2-yl]-2,5-diphenyltetrazolium bromide (MTT) assay kit according to the manufacturer’s instructions (Sigma-Aldrich, St. Louis, MO, USA). The formazan was dissolved in 100% dimethyl sulfoxide (DMSO) and measured spectrophotometrically at 540 nm using a Multiskan GO Microplate Spectrophotometer (Thermo Fisher Scientific, Waltham, MA, USA).

### 4.5. Western Blotting

Western blotting was performed as described previously [61]. Briefly, the total protein concentration was determined using the Bradford reagent (Bio-Rad, Hercules, CA, USA), and 20 μg of total proteins was separated using SDS-PAGE and transferred to PVDF membranes (Millipore, Bedford, MA, USA). The following antibodies were purchased from Cell Signaling Technology (Beverly, MA, USA) and used: AKT (1:1000, #4691), p-AKT (1:1000, #4060), Bax (1:1000, #2772), Bcl-2 (1:1000, #15071), Beclin1 (1:1000, #3495), Caspase-3 (1:1000, #9662), MLKL (1:1000, #14993), p-MLKL (1:1000, #91689), PARP (1:1000, #9542), p-PRAS40 (1:1000, #2997), p62 (1:1000, #5114), p-RB (1:1000, #8180), RIP (1:1000, #3493), p-RIP (1:1000, #65746), RIP3 (1:1000, #13526), pRIP3 (1:1000, #93654), p-STAT3 (1:1000, #9145), and Survivin (1:1000, #2808). β-actin (C4, 1:1000, #sc-47778), Cdk4 (1:1000, #sc-23896), Cdk6 (1:1000, #sc-7961), and CyclinD1 (1:1000, #sc-20044), were purchased from Santa Cruz Biotechnology (Santa Cruz, CA, USA). MMP13 (1:1000, NBP1-45723) was purchased from Novus Biologicals (Centennial, CO, USA). Protein signals were detected using the ProteinSimple detection system (ProteinSimple Inc., Santa Clara, CA, USA).

### 4.6. BrdU Incorporation Assay

A BrdU incorporation assay was performed to measure the quantification of DNA replication according to the protocol provided by the supplier using the colorimetric BrdU ELIZA Kit (Biovision, Milpitas, CA, USA).

### 4.7. Proteome Profiler Human Phospho-Kinase Array

Antibodies targeting human phosphokinases for capture and control were spotted in duplicates on the membranes. The following procedures were performed according to the protocol provided by the supplier (#ARY003C, R&D Systems, Minneapolis, MN, USA).

### 4.8. Autophagosome Formation Assay 

Autophagosome formation was performed using the DAPGreen Autophagy Detection Kit (Dojindo, Kumamoto, Japan) according to the manufacturer’s instructions. Images of autophagosomes were captured using an Olympus IX73 inverted microscope (Olympus Corporation, Tokyo, Japan) and an intravital multi-photon microscope system (IMPM) at the Korea Basic Science Institute (KBSI) (Gwangju, Republic of Korea). 

### 4.9. Cell Migration Assay

Cell migration was assessed using a wound-healing assay that was created by scratching the cell monolayer with a 200 μL pipette tip, as previously described [62]. After treatment with Vitex for 24 h and 48 h, images of the migrating cells were captured using a light microscope.

### 4.10. Cell Invasion Assay

The cell invasion assay was performed using a Boyden chamber with membranes coated with Matrigel solution (Corning Life Sciences, Tewksbury, MA, USA), as previously described [62]. After pretreatment with Vitex, cells were seeded and allowed to infiltrate for 4 h. Images of the invading cells were captured using a light microscope.

### 4.11. Statistical Analysis

Data were analyzed using GraphPad Prism version 5 (GraphPad Prism, Inc., San Diego, CA, USA). Data are presented as the mean with standard deviation (SD). Significance (*p* < 0.05) was evaluated using one-way analysis of variance with Dunnett’s post hoc test to compare all columns against the control column in the GraphPad Prism program.

## Data Availability

The data generated during the current study are available from the corresponding author upon reasonable request.

## Supplementary Materials

The supporting information can be downloaded at: https://www.mdpi.com/article/10.3390/ijms25073582/s1.

## Abbreviations

*A. apiacea*	*Artemisia apiacea* Hance ex Walp.
BrdU	Bromodeoxyuridine
MLKL	Mixed lineage kinase domain like pseudokinase
MMP13	Matrix metalloproteinase 13
MTT	3-[4:5-dimethylthiazol-2-yl]-2,5-diphenyltetrazolium bromide
PRAS40	40 kDa proline-rich Akt substrate
RIP	Receptor-interacting serine/threonine-protein kinase
Vitex	Vitexicarpin
