# Supplementary material for: Vitexicarpin Induces Apoptosis and Inhibits Metastatic Properties via the AKT-PRAS40 Pathway in Human Osteosarcoma
Source: Int J Mol Sci. 2024 Mar 22;25(7):3582. doi: 10.3390/ijms25073582 (PMC11012096; doi:10.3390/ijms25073582)

Supplementary Figure Legend

- **Supplementary Figure S1.** Effects of Vitex on STAT3 in the human osteosarcoma cells. Western blotting of phospho-STAT3 (p-STAT3) and β-actin levels. The amount of β-actin was detected as a loading control in the same sample.

Supplementary Fig. S1

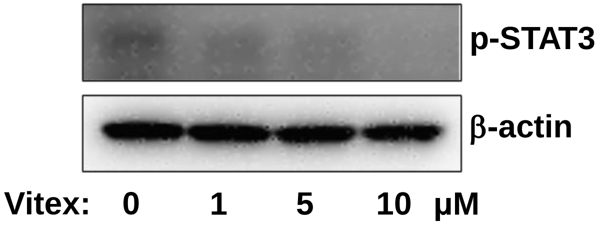

Supplement: Supplementary file 1 [file ijms-25-03582-s001.zip › ijms-2894849-supplementary.pdf]
